# Association of Methylentetraydrofolate Reductase (MTHFR) 677 C > T gene polymorphism and homocysteine levels in psoriasis vulgaris patients from Malaysia: a case-control study

**DOI:** 10.1186/1475-2891-11-1

**Published:** 2012-01-05

**Authors:** Siaw C Liew, Esha Das-Gupta, Shew F Wong, Nagarajah Lee, Najeeb Safdar, Adawiyah Jamil

**Affiliations:** 1 Department of Postgraduate Studies and Research, International Medical University, Kuala Lumpur, Malaysia; 2 Department of Internal Medicine, International Medical University, Seremban, Malaysia; 3 Open University, Kuala Lumpur, Malaysia; 4 Department of Dermatology, Tuanku Ja'afar Hospital, Seremban, Malaysia; 5 Department of Dermatology, Kuala Lumpur Hospital, Kuala Lumpur, Malaysia

## Abstract

**Background:**

The methylenetetrahydrofolate reductase (MTHFR) enzyme catalyzes the reduction of 5, 10-methylenetetrahydrofolate to 5-methyltetrahydrofolate and methyl donors. The methyl donors are required for the conversion of homocysteine to methionine. Mutation of MTHFR 677 C > T disrupts its thermostability therefore leads to defective enzyme activities and dysregulation of homocysteine levels.

**Methods:**

This case-control study (n = 367) was conducted to investigate the correlation of the MTHFR gene polymorphism [NM_005957] and psoriasis vulgaris amongst the Malaysian population. Overnight fasting blood samples were collected from a subgroup of consented psoriasis vulgaris patients and matched controls (n = 84) for the quantification of homocysteine, vitamin B_12 _and folic acid levels.

**Results:**

There was no significant increase of the MTHFR 677 C > T mutation in patients with psoriasis vulgaris compared with controls (*χ*^2 ^= 0.733, p = 0.392). No significant association between homocysteine levels and MTHFR gene polymorphism in cases and controls were observed (F = 0.91, df = 3, 80, p = 0.44). However, homocysteine levels in cases were negatively correlated with vitamin B_12 _(r = -0.173) and folic acid (r = -0.345) levels. Vitamin B_12 _and folic acid levels in cases were also negatively correlated (r = -0.164).

**Conclusions:**

Our results indicate that there was no significant association between the MTHFR gene polymorphism and psoriasis vulgaris in the Malaysian population. There was no significant increase of the plasma homocysteine level in the psoriasis patients compared to the controls.

## Background

Psoriasis vulgaris also known as chronic plaque psoriasis, is a common subtype of psoriasis that affects about 80% of psoriatic patients worldwide [1]. Psoriasis has a prevalence of 1-2% worldwide and it affects both sexes equally [2]. The incidence in Malaysia is about 2.15% [3]. Till date, the exact pathogenesis of this disease is still unclear. Many different postulated aetiological hypotheses have been forwarded to explain the pathogenesis of the disease, yet the more convincing evidences are being the genetic, environmental and immunological factors [4] . However, in the last two decades, the association between methylenetetrahydrofolate reductase (MTHFR) gene polymorphism and psoriasis vulgaris has attracted great interest amongst researchers worldwide. The contradicting results obtained from different studies conducted in the West and the Far East had further confounded the situation [5-7].

The MTHFR gene (NM_005957) is located at chromosome 1 (1p36.3). It catalyses the conversion of 5,10-methylenetetrahydrofolate to 5-methylenetetra-hydrofolate, which then leads to the remethylation of homocysteine to methionine [8,9]. This cycle is important for maintaining the methyl donors for DNA methylation, and hence gene regulation and cellular differentiation [5,10]. Polymorphism of the MTHFR gene involves the substitution of the nucleotide C with T at position 677 which leads to transition of Alanine to Valine. This reduces MTHFR enzymatic activity and thermostability, therefore interferes with the homocysteine levels [11,12]. The enzymatic remethylations of homocysteine to methionine by 5-methyltetrahydrofolate homocysteine methyltransferase rely heavily on the adequate levels of vitamin B_12 _and folic acid. Therefore, the deficiencies of the Vitamin B_12 _and folic acid will lead to hyperhomocysteinaemia and hypomethioninaemia [13-16].

MTHFR gene polymorphism has been reported to be associated with psoriasis vulgaris. Significant association of the polymorphism of the MTHFR gene and psoriasis vulgaris was reported in the Chinese population by Wang et al., 2000 [5]. This Chinese study was later refuted by the Austrian and the Czech study [6,7] on the Caucasian population. Therefore, we hypothesize that the MTHFR gene polymorphism 677 C > T was associated with psoriasis vulgaris in the Asian population and we tested our hypothesis on 200 Malaysian psoriasis vulgaris patients.

Recent studies also postulated hyperhomocysteinaemia as a risk factor for coronary heart disease, cerebrovascular accident and peripheral vascular diseases [17]. An increase in 5 μmol/L of homocysteine level was associated with significant increase risk of ischaemic heart disease, thromboembolism and stroke. A reduction of homocysteine level by 3 μmol/L with folic acid supplementation could reduce the risk significantly [18].

However to date, there is no other study that we are aware of which investigate the polymorphism of the MTHFR gene and psoriasis vulgaris on other ethnic groups of the Asian origin. We hypothesized that the functional polymorphism of the MTHFR gene might be associated with psoriasis vulgaris and hyperhomocysteinaemia. The purpose of this hospital-based case-control study was to determine the association of the MTHFR gene polymorphism and psoriasis vulgaris in the Malaysian population which comprises of the 3 major Asian populations: the Chinese, Indian and Malay ethnicities. This study also aim to investigate whether MTHFR 677 C > T polymorphism might play a role in the homocysteine level of the psoriasis vulgaris patients from Malaysia.

## Methods

### Study Population

The cases and controls were recruited from General Hospital Kuala Lumpur and Tuanku Ja'afar Hospital, Seremban, Malaysia from April 2009 to April 2010. All the psoriasis vulgaris patients, cases (n = 200) recruited were Malaysian with either Chinese, Indian or Malay ethnicity. The patients with psoriasis vulgaris were examined and diagnosed by the dermatologists based on clinical examination. The severity of the disease was then scored according to Psoriasis Area and Severity Index (PASI). A mixture of early and late onset of psoriasis vulgaris patients was included in this study (mean age 45.12 ± 14.20). The healthy controls (n = 167) were those without psoriasis vulgaris and were age- (± 5 years), gender- and ethnicity-matched with the respective patients. The samples were determined based on the criteria of 95% confidence and 85% statistical power in determining the confidence intervals of the parameters estimated in this study. The patients and controls were well informed about the nature of this study and their participations were voluntary. Informed consent was obtained from each subject. Approximately 5 mL of blood samples were withdrawn from each case or control. A subgroup (n = 41) of consented patients with homozygous CC, heterozygous CT and homozygous TT genotypes were subsequently selected or recruited for further study. Among the cases recruited (n = 200), only 41 cases consented for the subsequent skin biopsy procedure. The age- (± 5 years), gender- and ethnicity-matched healthy controls (n = 43) were those with homozygous CC, heterozygous CT and homozygous TT genotypes but without psoriasis vulgaris. Their blood samples were collected for the measurement of plasma homocysteine, vitamin B_12 _and folic acid levels after fasting for ~ 8 hours. Cases and controls with previous history of ischaemic heart disease, myocardial infarction, cerebrovascular accident, thromboembolic events were excluded from this study. The blood samples were sent to a commercial laboratory (Pathlab Malaysia Sdn Bhd) for the measurement of homocysteine, vitamin B_12 _and folate levels. The protocol of this study was designed according to the Declaration of Helsinki and was approved by the ethics committee of the International Medical University, Malaysia.

### Genotyping

**Figure 1:**
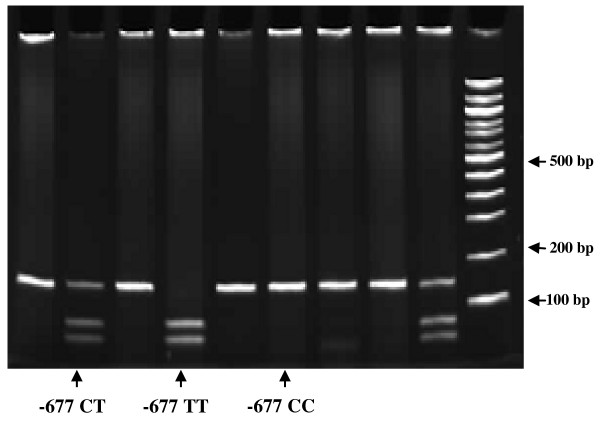
**Polyacrylamide gel (12%) electrophoresis analysis of the banding patterns of Taq-α1 digested MTHFR PCR-amplified products**.

**Figure 2:**
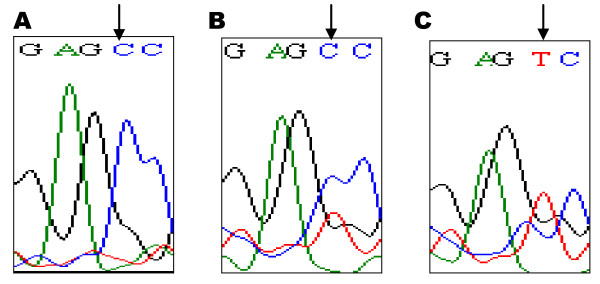
**DNA sequencing analysis of the MTHFR PCR-amplified products (NM_005957, rs1801133)**. Sequences of MTHFR 677 (A) homozygous CC, (B) heterozygous CT, and (C) homozygous TT.

DNA samples were extracted from the whole blood of the cases and controls using DNA extraction kit (Qiagen Inc, Germany) according to the manufacturer's protocol. The amount of the DNA was quantified and the quality of the DNA was determined. The MTHFR gene was amplified in a final volume of 25 μL using the following primers: 5'-GAA GCA GGG AGC TTT GAG GC-3' and 5'-CCC ATG TCG GTG CAT GCC TT-3' [5]. For each reaction, 12.5 μL of TopTaq master mix was used. Both forward (0.25 μL) and reverse (0.25 μL) primers were added. Finally, 2 μL of the extracted DNA was added into the mixture. PCR was performed under the following conditions: initial denaturation (3 minutes at 94°C), followed by a 3-step cycling processes (35 cycles) which consisted of denaturation (30 seconds at 94°C), annealing (30 seconds at 60°C) and extension (1 minute at 72°C); and a final extension at 72°C for 10 minutes. The final PCR product was 134 bp. The PCR products were digested with restriction enzyme Taqα1 (New England Biolabs, USA) and the digested products were separated, and visualized on 12% polyacrylamide gels using a BioVision 1000 gel documentation system (Vilber Lourmat, France). The typical polymerase chain reaction - restriction fragment length polymorphism (PCR-RFLP) was shown in Figure 1. The expected fragment sizes for the polymorphism are 59 bp and 76 bp. The DNA sequencing analysis of the MTHFR PCR amplified products (NM005957, rs1801133) were shown in Figure 2.

### Statistical Analysis

The association between the genotypes of the MTHFR polymorphism and allelic frequencies with the demographic variables was analyzed with Chi-square test. T-test was used to determine the association of homocysteine, vitamin B_12 _and folic acid levels between cases and controls irregardless of the genotypes. The association between the plasma homocysteine, vitamin B_12 _and folic acid levels and the MTHFR polymorphisms were analyzed using 3-way ANOVA. P values were considered statistically significant if they were less than 0.05. The sample size was determined using the power analysis, with the α error and β error set at 0.05 and 0.20 respectively. Approximately 40 subjects per group were sufficient for parametric test (Cohen, 1977) whilst for non-parametric test such as Chi-Square test, the expected frequency of a minimum 5 for each category was sufficient (Hays, 1994). The measure of skewness and kurtosis were used to assess the normality of the distribution of the parameters (homocysteine, vitamin B_12 _and folic acid levels).

## Results

### Demography of the subjects

In this study, great attention was given to the selection of the cases and the controls. The matching process was conducted to ensure that the cases and controls have similar demographic characteristics. Two hundred psoriasis vulgaris patients, diagnosed clinically by the dermatologists were age-, sex- and ethnicity- matched with 167 healthy controls. Table 1 showed the demographic characteristics of the cases and controls. Cases and controls with medical history of ischaemic heart disease, cerebrovascular accident or thromboembolic events were excluded from this study. Psoriatic Area and Severity Index (PASI) was performed on all psoriatic patients upon recruitment.

**Table 1:** Demographic characteristics of psoriasis vulgaris cases and healthy controls

Demography	Cases (n = 200)	Controls (n = 167)
Males, n (%)	113 (56.50)	87 (52.10)
Females, n (%)	87 (43.50)	80 (47.90)
Age, (mean ± SD)	45.12 ± 14.20	43.17 ± 14.66
PASI score (mean ± SD)	8.92 ± 7.64	N/A
		
Chinese, n (%)	39 (19.50)	33 (19.76)
Indian, n (%)	67 (33.50)	57 (34.13)
Malay, n (%)	94 (47.00)	77 (46.11)

### MTHFR 677 C > T gene polymorphism in cases and controls

The distribution of the MTHFR gene polymorphism amongst cases and controls are shown in Table 2. Genotype CC was found in 79.50% of cases whilst 20.50% were with genotype CT. As for the controls, 74.85% of the controls were with genotype CC, 23.95% were with genotype CT and 1.20% were with genotype TT. No significant difference in the genotypic distribution between cases and controls was observed (*χ*^2 ^= 0.733, p = 0.392). The occurrence of genotype CT was higher amongst cases compared with controls (adjusted odds ratio (OR) 1.11; 95% confidence interval (CI) 0.47 - 2.60). However this was not statistically significant (p = 0.812). The genotype TT was not detected in the cases but was found in two controls as such no further statistical analysis was performed. The frequencies of distribution of the T allele in cases and controls were comparable. No statistically significant difference in the frequency of allelic distribution between cases and controls was seen (*χ*^2 ^= 1.52, p = 0.218).

**Table 2:** Distribution of cases and controls according to races, genotypes and allelic frequencies of the MTHFR gene

MTHFR C > T	Cases (n = 200)	Controls (n = 167)	* P-value	**OR (95% CI)
CC, n (%)	159 (79.50)	125 (74.85)	p = 0.392	1.11 (0.47-2.60)
Chinese, n (%)	30 (18.87)	21 (16.80)	χ^2 ^= 0.733	
Indian, n (%)	57 (35.85)	49 (39.20)		
Malay, n (%)	72 (45.28)	55 (44.00)		
				
CT, n (%)	41 (20.50)	40 (23.95)		
Chinese, n (%)	9 (21.95)	11 (27.50)		
Indian, n (%)	10 (24.39)	22 (55.00)		
Malay, n (%)	22 (53.66)	7 (17.50)		
				
TT, n (%)	0 (0.00)	2 (1.20)		
Chinese, n (%)	0 (0.00)	1 (50.00)		
Indian, n (%)	0 (0.00)	1 (50.00)		
Malay, n (%)	0 (0.00)	0 (0.00)		
				
**ALLELE**				
C (%)	359 (89.75)	290 (86.83)	p = 0.218	
T (%)	41 (10.25)	44 (13.17)	χ^2 ^= 1.520	

* P-values obtained for the χ^2 ^test for the genotypes distribution or allelic frequencies between cases and controls.

** OR is the risk of genotype CT in cases compared to controls

The association between MTHFR gene polymorphism (CC and CT) and ethnicities (Chinese, Malay and Indian) was analyzed. The frequencies of the genotype CT were significantly higher in the Malay population (p < 0.002) in the cases compared with controls. The Chi-square test for Goodness-of-Fit was used, since each cell has an expected frequency of at least 5 subjects. Table 2 summarizes the genotype frequencies and the Chi- square test results. Subjects with TT polymorphism were not included since the numbers were too few.

### Association between the plasma homocysteine, vitamin B_12 _and folic acid levels in cases and controls

The plasma homocysteine, vitamin B_12 _and folic acid levels of the selected cases (n = 41) and controls (n = 43) were summarized in Table 3. Homocysteine, vitamin B_12 _and folic acid levels of cases regardless of the MTHFR gene genotype were compared with the controls. There was no significant difference between these two groups on all the three parameters assessed with t-test analysis (t = 0.955, p = 0.342 for homocysteine; t = 1.574, p = 0.119 for vitamin B; t = -0.745, p = 0.459 for folic acid). The cases with genotype CT had the highest homocysteine levels, followed by controls with genotype CC, cases with genotype CC and controls with genotype CT. No significant association between homocysteine levels and MTHFR gene polymorphism in cases and controls were seen (F = 0.91, df = 3, 80, p = 0.44). Similarly, no significant association between vitamin B_12 _levels and MTHFR gene polymorphism in both cases and controls was seen (F = 1.34, df = 3, 80, p = 0.27). There was no significant association between folic acid levels and MTHFR gene polymorphism in cases and controls (F = 1.34, df = 3, 80, p = 0.27; Table 3).

**Table 3:** Demographic distribution of cases and controls according to their genotypes and homocysteine, vitamin B_12 _and folic acid levels

	Cases (CC) (n = 19)	Cases (CT or TT) (n = 22)	Controls (CC) (n = 21)	Controls (CT or TT) (n = 22)	*P-value
**Males, n (%)**	**11 (42.10)**	**11 (50.00)**	**10 (47.60)**	**11 (50.00)**	
Chinese	3 (27.27)	3 (27.27)	2 (20.00)	2 (18.18)	
Indian	2 (18.18)	2 (18.18)	2 (20.00)	4 (36.36)	
Malay	6 (54.55)	6 (54.55)	6 (60.00)	5 (45.45)	
					
**Females, n (%)**	**8 (42.10)**	**11 (50.00)**	**11 (52.38)**	**11 (50.00)**	
Chinese	2 (25.00)	3 (27.27)	3 (27.27)	4 (36.36)	
Indian	3 (37.50)	5 (45.45)	5 (45.45)	4 (36.36)	
Malay	3 (37.50)	3 (27.27)	3 (27.27)	3 (27.27)	
					
Age, mean ± SD	38.41 ± 12.36	45.91 ± 13.17	43.43 ± 11.93	43.64 ± 10.84	
PASI score, mean ± SD	9.22 ± 10.85	6.37 ± 4.97	N/A	N/A	
Homocysteine (μmol/L)	14.92 ± 4.13	16.31 ± 4.56	15.42 ± 3.81	14.39 ± 3.59	[p = 0.440]
Vitamin B_12 _(pg/mL)	482.22 ± 156.98	465.84 ± 210.73	396.38 ± 96.06	444.20 ± 112.19	[p = 0.270]
Folic acid (ng/mL)	7.66 ± 3.91	7.24 ± 4.61	6.62 ± 3.19	9.14 ± 6.40	[p = 0.270]

* P-values obtained for the F-test for the homocysteine, vitamin B_12 _and folic acid levels in cases and controls with different genotypes.

The correlation between homocysteine, vitamin B_12 _and folic acid levels in cases and controls were determined. Homocysteine levels in cases were negatively correlated with vitamin B_12 _(r = -0.173) and folic acid (r = -0.345) levels. Similarly, vitamin B_12 _and folic acid levels in cases were negatively correlated (r = -0.164). Homocysteine levels in controls were negatively correlated with vitamin B_12 _(r = -0.439) and folic acid (r = -0.598) levels. However, vitamin B_12 _and folic acid levels in controls were positively correlated (r = 0.250).

The homocysteine, vitamin B_12 _and folic acid levels in cases and controls were summarized in Table 4 according to gender and ethnicity. The associations of gender and ethnicity with homocysteine, vitamin B_12 _and folic acid levels were analyzed with Three Way ANOVA. Gender was the only variable that was significantly associated with homocysteine levels (F = 19.538, p = 0.000). Males (mean = 17.00 and SD = 4.06) have significantly (p = 0.001) higher homocysteine levels compared with female (mean = 12.43 and SD = 3.10). It can be concluded that plasma homocysteine levels were not associated with psoriasis vulgaris, MTHFR gene polymorphism and ethnicity.

**Table 4:** Homocysteine, vitamin B_12_, and folic acid levels for cases and controls according to gender and ethnicities

	Cases (CC) (n = 19)	Cases (CT or TT) (n = 22)	Controls (CC) (n = 21)	Controls (CT or TT) (n = 22)
**Homocysteine**, (Normal reference value = 5.0-15.0 μmol/L)				
**Males**, mean (μmol/L)				
Chinese	14.97	17.67	13.68	12.25
Indian	13.25	19.85	19.50	16.13
Malay	18.05	17.80	15.85	16.66
				
**Females**				
Chinese	12.35	13.20	13.80	13.55
Indian	13.47	16.28	13.14	13.15
Malay	13.23	12.77	12.40	11.93
				
**Vitamin B_12_**, (Normal reference value = 211-911 pg/mL)				
**Males**, mean (pg/mL)				
Chinese	495.0	271.5	271.5	483.5
Indian	630.5	315.0	317.0	373.3
Malay	410.8	562.7	431.5	485.0
				
**Females**				
Chinese	385.0	386.3	478.0	420.7
Indian	452.0	386.0	414.4	420.0
Malay	620.7	625.3	350.7	513.0
				
**Folic acid**, (Normal reference value = > 2.8 ng/mL)				
**Males**, mean (ng/mL)				
Chinese	11.8	14.9	5.2	14.1
Indian	7.5	6.1	3.6	10.5
Malay	4.7	5.5	6.0	5.7
				
**Females**				
Chinese	11.5	24.0	12.5	11.3
Indian	12.5	9.0	13.4	7.8
Malay	6.1	7.1	7.5	13.8

As for vitamin B_12_, similar results were seen as of homocysteine levels. Nonetheless vitamin B_12 _levels were associated with gender.

Both cases and controls of the Chinese ethnicity had significantly (p = 0.000) higher folic acid levels (mean = 11.51, SD = 6.10) compared with Indians (mean = 7.35, SD = 4.92) and Malays (mean 6.10, SD = 2.37). However, folic acid levels in different ethnic groups were not associated with gender (p = 0.062) and MTHFR gene (p = 0.128).

## Discussion

The association between MTHFR gene polymorphism and psoriasis vulgaris was reported by Wang et al. (2000) [5] and was refuted by the studies conducted in Austria (Weger et al., 2008) [6] and the Czech Republic (Vasku et al., 2009) [7]. These contradictory findings were postulated to the probable differences in ethnicities. Till date, no other report on the prevalence of this MTHFR gene polymorphism and psoriasis vulgaris was documented for the Asian population. The association of the MTHFR gene polymorphism and psoriasis vulgaris has been postulated due to the higher risk of psoriasis patients developing cardiovascular and cerebrovascular complications. Nonetheless, the pathogenesis is not known till date [5-7,17,18].

The association of the MTHFR gene polymorphism and psoriasis vulgaris in the Malaysian population i.e. Chinese, Indian and Malay ethnic groups was investigated in this case-control hospital based study. Two hundred patients and 167 controls were recruited. The methodology of this study was conducted according to Wang et al. (2000) [5]. Our results demonstrated that there was no statistically significant association between MTHFR C > T gene polymorphism and psoriasis vulgaris in the Malaysian population, which consistent with the Austrian [6] and the Czech Republic's [7] findings on Caucasian population. Our study thus contradicts the published study in China [5]. Our study also investigated the association of the MTHFR gene polymorphism and ethnicities in Malaysia namely the Chinese, Indian and Malay. We reported that there were no significant association between MTHFR gene polymorphism in psoriasis vulgaris and ethnicities in the Malaysian population. Though a larger sample size of patients was investigated in the present study, we did not find any significant association of the MTHFR gene polymorphism and psoriasis vulgaris as compared with the previous Chinese study on only 39 Chinese patients [5].

The prevalence of the MTHFR 677 C > T is variable depending on the geography and ethnicity [19]. Botto et al. (2000) reported that the worldwide T allele frequency was highest in the Italian and the Hispanics, and lowest in the American Blacks and sub-saharan Africa. In the European population, the distribution of the homozygous T allele was highest in the Italian and lowest in the German [20-22]. In the Blacks, the frequency of T homozygosity in the sub-Saharan African population showed zero percentage [23,24]. In Asian, limited data could be retrieved as only the Japanese population was studied and the population percentage of the homozygous T mutation was 11% [25,26]. An interesting finding that we discovered in this study was the homozygosity of the T genotypes was found only in 2 of the controls but not in the cases. This finding contradicted the Chinese study [5] which reported the significant increased of homozygous TT in the cases compared with controls (p < 0.05). We hypothesized this variation may be due to the geographical differences as seen in the Europeans [27].

The MTHFR gene polymorphism was reported to be associated with hyperhomocysteinaemia which has deleterious effects on the cardiovascular system [13,28]. Hence, psoriasis vulgaris patients with MTHFR gene polymorphism may be at greater risk of cardiovascular diseases and thromboembolic events. Therefore, we investigated the relationship between MTHFR gene polymorphism and homocysteine, vitamin B_12 _and folic acid levels in selected 41 cases and 43 controls. We demonstrated that there was no significant relationship between the MTHFR gene polymorphism and homocysteine, vitamin B_12 _or folic acid levels in this subset of subjects.

The relationship of homocysteine, vitamin B_12 _and folic acid levels compared with the ethnicities and the MTHFR gene polymorphism was analyzed using 3-way ANOVA. We did not find any statistically significant relationship amongst these groups. Nonetheless, males irregardless of the polymorphism of the MTHFR gene have a higher homocysteine levels when compared to the females (p = 0.001). Overall, the Indian males and Malay males have higher homocysteine levels than the Chinese males and a significantly lower folic acid level compared to the Chinese (p = 0.000).

In Malaysia, the dietary intakes varied amongst the ethnic groups and also varied amongst the genders [29,30]. The Indian and the Malay groups have a higher intake of coconut oil, processed sugars and condensed milk in their diet when compared to the Chinese. We proposed that the significant increased in homocysteine level in the Indian and Malay males might be due to the different dietary intakes between different ethnic groups amongst the males. The observation that the folic acid level that is significantly higher in the Chinese again pinpointed the beneficial effect of folic acid in the lowering of the plasma homocysteine level [12]. Therefore, these differences might be a beneficial consideration to be taken note of when other epidemiological studies are being carried out in these populations in the future.

The limitation of this study was the centrality of the recruitment of the subjects in the area of Kuala Lumpur and Seremban and we did not recruit subjects from the northern, eastern or southern states of Malaysia. Perhaps, there would have been a difference in the genotypes distribution in these places compared with the ones we have recruited. Future studies might be warranted to investigate the variation of the MTHFR 677 C > T gene geographically, perhaps on other Asian populations using a bigger sample size and a larger population. The inclusion of other single nucleotide polymorphism of the MTHFR gene would be beneficial to investigate the association of the presence of synergistic effects of the haplotypes and psoriasis vulgaris.

This study has again pinpointed the independent hyperhomocysteinaemia irrespective of the possession of psoriasis vulgaris, mutation of the MTHFR gene and its prevalence in the male gender. With the prior knowledge of hyperhomocysteinaemia as a risk for cardiovascular and thromboembolic events, routine homocysteine and folic acid screening should be included as part of psoriasis vulgaris and cardiovascular disease management. The routine supplementation of folic acid in the diet of the psoriasis vulgaris patients could be of benefit to them.

## Conclusions

In summary, we concluded that the MTHFR 677 C > T gene polymorphism and the T allele occurrence were not significantly associated with psoriasis vulgaris in the Malaysian population. We also concluded that there was no significant association between the plasma homocysteine levels and psoriasis vulgaris with or without the MTHFR gene polymorphism.

## List of abbreviations

**bp**: base pair; **DNA**: deoxyribose nucleic acid; **PCR**: polymerase chain reaction; **RFLP**: reaction fragment length polymorphism; **SD**: standard deviation; **SDS**: sodium dodecyl sulphate; **SDS-PAGE**: sodium dodecyl sulphate polyacrylamide gel electrophoresis; **U**: unit; **Val**: Valine; **V**: volt; **vs**.: versus.

## Competing interests

The authors declare that they have no competing interests.

## Authors' contributions

SCL participated in the conception of the study, the inception of the study design, carried out the patients sampling, molecular genetic study (DNA extraction, PCR, RFLP) and drafting of the manuscript. EDG, SFW, NS, AJ participated in the conception of the study, the inception of the study design, carried out the patients sampling and drafting of the manuscript. N Lee participated in the conception of the study, the inception of the study design and performed the statistical analysis. All authors read and approved the final manuscript.

## Authors' information

S.C.L is a medical doctor and is currently pursuing her Master of Science (MSc) at the International Medical University. This research paper covered part of her MSc project. E.D.G, S.F.W and N.S are her supervisors for this project. N.L is the statistician overseeing this project and A.J is the co-investigator for this project.
